# Prospective Assessment of Carboxyhemoglobin and Methemoglobin Levels in Operating Room Personnel During HIPEC/Peritonectomy Compared with Standard Colorectal Surgery

**DOI:** 10.3390/jcm15176711

**Published:** 2026-08-29

**Authors:** Emil Kinda, Petar Matosevic, Sanda Smud Orehovec, Ivan Romic, Rudolf Radojkovic, Branko Bogdanic

**Affiliations:** 1 Department of Surgery, University Hospital Center Zagreb, 10000 Zagreb, Croatia; 2 Medical School Zagreb, University of Zagreb, 10000 Zagreb, Croatia

**Keywords:** smoke, peritonectomy, safety, carbon monoxide

## Abstract

**Background/Objectives**: In the operating room, surgical staff are exposed to inhalation of smoke from the combustion of tissues when using electrocautery. By introducing HIPEC/peritonectomy in the treatment of malignant neoplasms of the peritoneum, exposure to smoke is significantly increased. To adequately perform this procedure, high-power monopolar cautery is used. The aim of this study was to assess carbon monoxide exposure associated with surgical smoke generated during peritonectomy procedures and to compare it with exposure during standard colorectal resections. **Methods**: In this prospective observational study, a total of 360 blood samples were collected and analyzed from surgeons participating in surgical procedures. The study cohort was divided into two groups: the HIPEC/peritonectomy group and the standard resection group. Blood samples were obtained from three surgeons who participated in a total of 30 procedures, comprising 15 HIPEC/peritonectomy procedures and 15 standard resection procedures. Samples were collected at the start of the intervention, at the 20th and 40th minutes, and at the end of procedures. **Results**: COHb values increased over time, and levels were higher in the peritonectomy group. An increase in MetHb levels was seen in HIPEC/peritonectomy, but not in the standard colorectal resection group. The observed values did not lead to symptoms of acute CO poisoning. **Conclusions**: The results suggest that the use of electrocautery increases carboxyhemoglobin (COHb) and methemoglobin (MetHb) levels in surgical staff; however, these increases did not reach clinical significance. The long-term health consequences of repeated exposure to surgical smoke, as well as the effectiveness of personal protective equipment and smoke evacuation systems in reducing this exposure, should be investigated in future studies.

## 1. Introduction

Exposure to surgical smoke is unavoidable in the daily work of operating room staff. Surgical smoke is generated by the incomplete combustion of proteins and lipids during the use of electrosurgical devices for cutting and coagulating tissue. It primarily contains smaller particles that are associated with thermal and chemical tissue injury. Carbon monoxide (CO) is one of the constituents of surgical smoke and may lead to an increase in COHb levels in both patients and surgical staff [1].

The introduction of novel therapeutic procedures and the emergence of technological advancements in some cases may bring new risks for medical personnel, especially for operating room personnel (ORP). ORP are exposed to smoke on a daily basis mostly as a result of smoke production during electrocautery or use of tissue sealing devices. ORP and professionals involved in occupational health and safety are increasingly aware of the negative impact of surgical smoke on ORP health [2,3]. This awareness was significantly triggered by the COVID-19 crisis and research during this period, which contributed to understanding of surgical smoke issues and risks associated with it [4]. However, there is still a limited amount of data related to surgical smoke-related risks for specific surgical procedures. In studies, it is important to evaluate chemical compounds, plume particle concentrations, and the effect of electrocautery settings on smoke production.

Significant advances have been achieved in the treatment of many diseases, including peritoneal carcinomatosis. In the past, this condition was considered incurable, and patients were limited to symptomatic treatment. Cytoreductive surgery has since become the gold standard in the management of peritoneal carcinomatosis. This therapeutic approach has resulted in better survival outcomes and an improvement in patients’ quality of life [5].

However, it remains unclear what long-term effects this technically demanding and lengthy procedure may have on the health of surgeons and other operating room personnel. At the Department of Surgery, University Hospital Centre Zagreb, this method was first introduced in 1997. Over a ten-year period, 160 peritonectomy procedures combined with hyperthermic intraperitoneal chemotherapy (HIPEC) were performed. During peritonectomy procedures, operating room personnel are exposed to a variety of occupational hazards, including exposure to cytotoxic agents. It is characterized by prolonged and intensive use of high-energy electrocautery since parietal peritoneum must be extensively excised along with visible carcinomatous lesions. As a result, surgical staff are exposed to larger amounts of smoke when compared to less extensive or shorter surgical procedures.

CO is one of the main components of surgical smoke. It is produced by incomplete combustion of hydrocarbons. CO is a colorless, odorless, non-irritating gas, so its presence often goes unnoticed. Carbon monoxide poisoning can be either acute or chronic [6].

Concentrations of CO in the air starting from 100 ppm can have harmful effects on health, as it may lead to hypoxic stress in healthy individuals due to its high affinity for hemoglobin. The inhaled carbon monoxide rapidly diffuses into the blood through the alveolar-capillary membrane in the lungs. About 80% of CO binds to hemoglobin, forming COHb and methemoglobin, while the remaining amount of CO is distributed in tissues. The mechanisms of CO toxicity are not fully clarified. CO displaces oxygen molecules from hemoglobin because its binding affinity for hemoglobin is 220 times greater than that of oxygen. A stable complex of CO and hemoglobin forms in erythrocytes. This causes a leftward shift of the oxygen dissociation curve (reducing oxygen release from hemoglobin to tissues) and inhibits mitochondrial respiration [7]. To our knowledge, there are no studies specifically investigating the effects of CO inhalation during the HIPEC procedure.

In this comparative study, we aimed to evaluate the levels of COHb and MetHb in venous blood of ORP during HIPEC/peritonectomy and standard colorectal resections. We hypothesized that HIPEC/peritonectomy was associated with higher venous blood COHb and MetHb levels.

## 2. Materials and Methods

The study was conducted prospectively at the Department of Surgery, University Hospital Centre Zagreb, over a period of 40 months (from September 2012 to January 2016). A total of 360 blood samples from surgeons doing procedures were analyzed, divided into two groups: HIPEC-peritonectomy group (HPG) and standard colorectal resection group (SCRG). Samples from 3 surgeons who participated in the procedures, a total of 30 procedures, 15 in each group, were collected. Samples were collected at the start of the intervention, at the 20th and 40th minutes, and at the end of procedures.

Regarding the patient eligibility criteria, SCRG included patients requiring curative major colorectal resections (hemicolectomies, rectal resections, or transverse colon resection) without signs of peritoneal carcinomatosis. HPG included patients with abdominal tumors and confirmed peritoneal carcinomatosis for whom HIPEC/peritonectomy with primary tumor resection was indicated. Patients with recurrent disease, those undergoing palliative procedures, or those aged <18 years were excluded from both groups. Regarding the surgical participants, to minimize selection bias, we ensured that the same operator and first and second assistants were present during each procedure. All participating surgeons were non-smokers, had no known pulmonary disease, and no obvious occupational or private-life sources of CO exposure were identified. The primary outcome was the analysis of baseline levels and changes in COHb and MetHb levels over the course of the operation in surgeons. Secondary outcomes included comparison of COHb and MetHb changes between the two groups; and analysis of COHb and MetHb level changes for each member of the surgical staff throughout the operative period.

For all procedures, a high-power monopolar electrosurgery (120–200 W) on a pure coagulation waveform was used. Electrical energy was generated by the ForceTriad™ Energy Platform (Valleylab, Tyco Healthcare Group LP, Boulder, CO, USA). Disposable electrosurgical pencils with interchangeable electrodes were employed, including blade electrodes, extended blade electrodes (16 cm), ball electrodes (5 mm), and extended ball electrodes (13 cm, 5 mm). All surgical procedures were performed in the abdominal surgery operating theatre at the University Hospital Centre Zagreb. The operating room was equipped with a laminar airflow system providing 23 air exchanges per hour. The floor area of the operating room was 42 m^2^, with a total volume of 126 m^3^. The mean relative humidity was 32.2%, and the mean ambient temperature was 21.5 °C. Operating room personnel wore Meditex surgical masks (R1144). Surgical smoke was evacuated using the OptiMumm™ Smoke Evacuator (Valleylab, Tyco Healthcare Group LP, Boulder, CO, USA). Smoke evacuators were used consistently throughout all procedures, and the distance between the suction inlet and the source of the surgical smoke was no more than 5 cm.

Exposure to inhaled carbon monoxide was assessed indirectly by measuring COHb and MetHb levels in heparinized venous blood samples. Measurements were performed using a Rapidlab 1265 blood gas analyzer (Siemens, Munich, Germany) and a GEM OPL co-oximetry analyzer (Instrumentation Laboratory, Bedford, MA, USA). The analyzer employs co-oximetry (spectrophotometry) to determine total hemoglobin concentration and hemoglobin fractions, including oxyhemoglobin, deoxyhemoglobin, MetHb, and COHb.

Venous blood samples from the primary surgeon, first assistant, and second assistant were collected at regular 20-min intervals (baseline, 20 min, 40 min, and a final sample at the end of each procedure). Heparinized venous blood was used for the determination of COHb and MetHb concentrations. Blood samples were collected using balanced-heparin syringes manufactured by Siemens (Munich, Germany). Following collection, each sample was gently mixed for approximately 20 s and immediately analyzed using the Rapidlab 1265 analyzer (Siemens Healthineers, Munich, Germany). The reference ranges were as follows: MetHb < 1.5% and COHb < 5.0%. Before the start of each surgical procedure, a peripheral venous cannula was inserted into the primary surgeon, first assistant, and second assistant using a standardized venipuncture technique. The minimal required blood sample volume was 1 mL.

### Statistical Analysis

An a priori sample size calculation was performed based on the anticipated difference in the change in COHb levels from baseline to the end of the surgical procedure for colorectal resections. Assuming a two-sided significance level of 0.05, a statistical power of 80%, equal allocation between groups, an anticipated between-group difference of 0.20% in COHb change, and a standard deviation of 0.18%, the standardized effect size was estimated at 1.11. Based on these assumptions, a minimum of 14 participants per group was required. The target sample size was rounded to 15 participants per group, resulting in a total planned sample of 30 participants. Categorical data are presented using frequencies and numerical data using the mean and standard deviation for normally distributed data and the median and interquartile range for non-normally distributed data. The Shapiro-Wilks test was used for normality analysis. Differences in numerical variables that did not follow a normal distribution between two independent groups were tested using the Mann–Whitney U test. Differences in normally distributed numerical variables between more than two measurements were tested with the Friedman test, while the Wilcoxon signed-rank test was used in cases of two measurements. In addition, longitudinal COHb and MetHb measurements were analyzed using a linear mixed-effects model. Treatment group (Standard vs. Peritonectomy), time (0, 20, 40 min, and end of observation), and the treatment-by-time interaction were specified as fixed effects. The significance level was set at *p* = 0.05. Data were analyzed using MedCalc Statistical Software version 13.1.2 (MedCalc Software bvba, Ostend, Belgium; http://www.medcalc.org; accessed on 4 February 2024).

## 3. Results

Demographic data

Patients were divided into two groups, with 15 patients undergoing HIPEC/peritonectomy and the other 15 undergoing standard colorectal resection. Data on age and sex are presented in Table 1. Patients undergoing standard colorectal resections were significantly older than those undergoing HIPEC/peritonectomy (median age, 64 vs. 56 years; *p* = 0.020). Overall, there were 12 male and 18 female patients; however, there was no statistically significant difference in sex distribution between the groups (Fisher’s exact test; *p* = 0.060). The HIPEC/peritonectomy group comprised 15 procedures performed in patients diagnosed with ovarian carcinoma (7 procedures), colorectal carcinoma with peritoneal dissemination (2 procedures), pseudomyxoma peritonei (5 procedures), and gastric adenocarcinoma with peritoneal dissemination (1 procedure). Group B comprised 15 standard colorectal resection procedures: 7 rectal resections, 4 right hemicolectomies, 2 left hemicolectomies, and 2 sigmoid resections.

### 3.1. COHb and MetHb in Surgeons with Regard to the Type of Surgery

**Figure 1:**
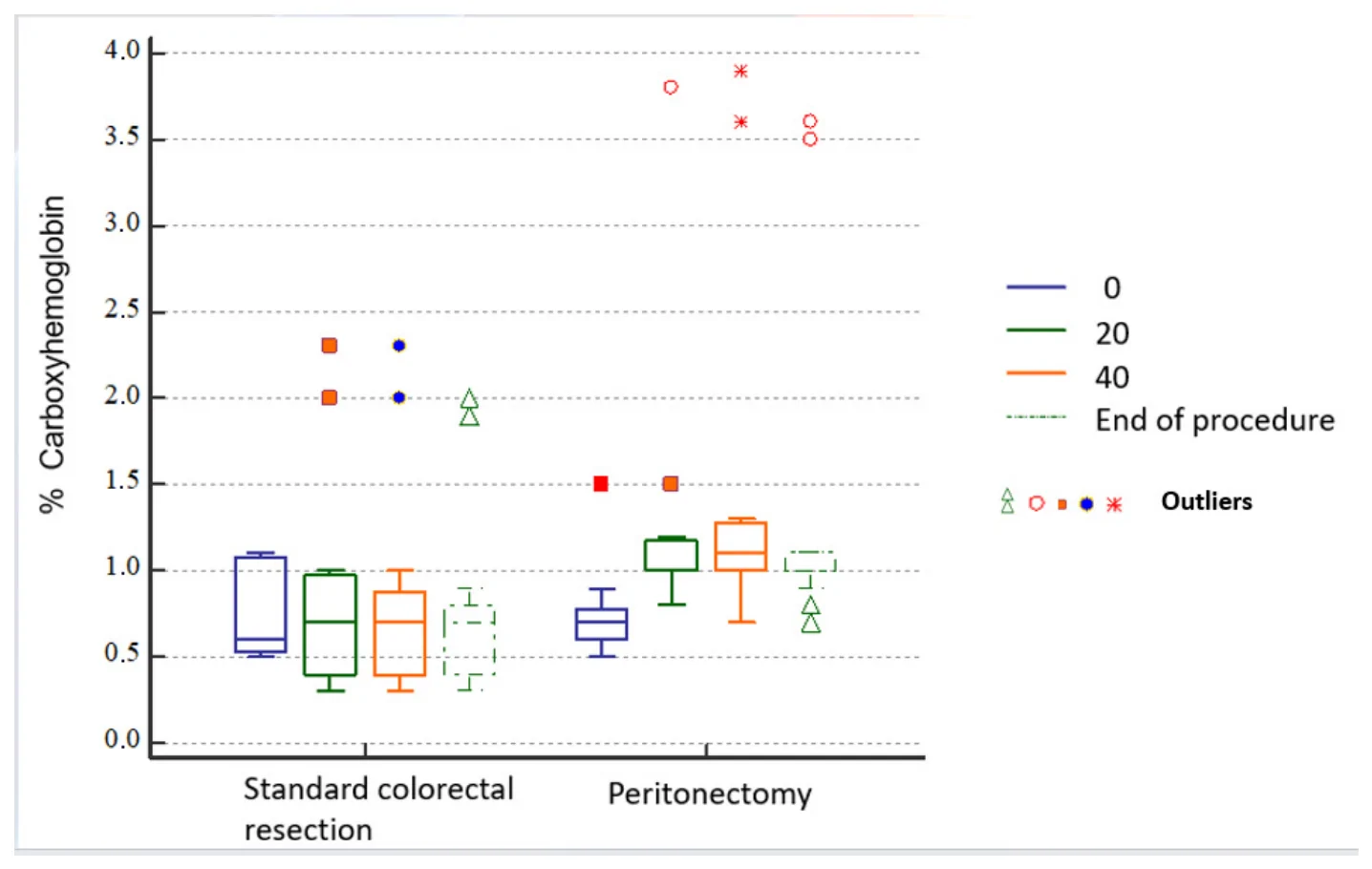
COHb in studied groups with different time points of the procedure.

Blood COHb and MetHb levels of all three surgeons were measured at four timepoints. Results are demonstrated in Table 2. There was an increase in COHb levels that was comparable at the beginning of the procedure, but it differed in all later timepoints since HIPEC/peritonectomy resulted in higher levels (Table 2, Figure 1).

Conversely, as Table 3 shows, MetHb levels were higher in the standard resection group in all time points, including preprocedural measurements.

### 3.2. COHb i MetHb Levels According to Surgical Procedure Type and Surgeon

#### 3.2.1. Results for Primary Surgeon

There was no difference in the COHb levels at the beginning of the procedure between the two groups (Table 4). The difference was noted at the 20th and 40th minutes and at the end of the procedure. Baseline MetHb levels were higher in the SCRG, and results did not demonstrate significant MetHb level increase in either group. Rather, it demonstrates higher MetHb values in the SCRG at each time point. Interestingly, MetHb even decreased over time in the SCRG group when comparing baseline and final results (Table 3 and Table 5).

#### 3.2.2. Results for First Assistant

Identical analysis was performed for the first assistant. There was no difference in the COHb levels at the beginning of the procedure. The difference was noted at the 20th and 40th minutes and at the end of the procedure. The COHb levels were higher in the peritonectomy group, and MetHb values were comparable to the primary surgeon group (Table 6 and Table 7).

#### 3.2.3. Results for Second Assistant

In second assistants, COHb levels were higher over time as well, and values differed between the groups, as can be seen in Table 8. However, in this case there was also a statistically significant difference in MetHb levels between HPG and SCRG (Table 9).

### 3.3. Analysis of COHb and MetHb Levels According to the Duration of Standard Colorectal Resection

In standard colorectal resections, a statistically significant difference was found between all evaluated time points except between 0 and 20 min (Table 10).

A less pronounced difference was seen in MetHb levels, but there was still a significant difference between 20 min and the end of the procedure (Table 11).

### 3.4. COHb and MetHb Analysis According to Peritonectomy Duration

A statistically significant difference was observed in the COHb levels between the preoperative measurement and those obtained at 20 min, 40 min, and at the end of the procedure. In addition, statistically significant increases were observed between the 20th and 40th minute measurements and between the 40th minute and the end-of-procedure measurement (Table 12).

A statistically significant difference was observed in the MetHb levels according to the duration of the procedure. Significant differences were found between the baseline measurements obtained at the beginning of the procedure and those recorded at 20 min, 40 min, and at the end of the procedure (Table 13).

A statistically significant greater increase in blood COHb levels was observed in surgeons performing peritonectomy procedures compared with those performing standard colorectal resections (Table 14 and Table 15). Differences were observed for MetHb levels as well.

The linear mixed-effects model demonstrated a significant group-by-time interaction for COHb levels, indicating significantly different COHb trajectories between the two groups (Table 16). In the HPG, COHb levels increased significantly from baseline by 0.333% at 20 min (95% CI, 0.291–0.376; *p* < 0.001), 0.480% at 40 min (95% CI, 0.438–0.522; *p* < 0.001), and 0.380% at the end of the observation period (95% CI, 0.338–0.422; *p* < 0.001). In contrast, no significant change was observed in the SCRG at 20 min (β = 0.007%, 95% CI, −0.036 to 0.049; *p* = 0.758), while COHb levels were significantly lower than baseline at 40 min (β = −0.073%, 95% CI, −0.116 to −0.031; *p* < 0.001) and at the end of observation (β = −0.100%, 95% CI, −0.142 to −0.058; *p* < 0.001).

At baseline, COHb concentrations did not differ significantly between groups (0.76 ± 0.30% vs. 0.77 ± 0.14%; *p* = 0.928). However, significant between-group differences emerged at 20 min and persisted at 40 min and at the end of the procedure, with higher COHb concentrations observed in the HPG at all subsequent time points (all *p* < 0.001).

The linear mixed-effects model demonstrated a significant group-by-time interaction for MetHb concentrations (Table 17). Compared with the HPG, the SCRG showed significantly lower changes in MetHb at 20 min (β = −0.148, 95% CI −0.179 to −0.117; *p* < 0.001), 40 min (β = −0.201, 95% CI −0.232 to −0.170; *p* < 0.001), and at the end of observation (β = −0.191, 95% CI −0.222 to −0.160; *p* < 0.001). These findings indicate significantly different MetHb trajectories between the two surgical groups over the course of the procedure.

## 4. Discussion

The aim of this study was to assess the exposure of operating room personnel to harmful occupational factors, primarily carbon monoxide. Current evidence is primarily based on documented individual cases and laboratory experiments. However, caution should be exercised when interpreting the results of in vitro experimental studies and extrapolating them to conditions encountered in everyday clinical practice [8]. Organizations responsible for occupational health and workplace safety have not yet established comprehensive recommendations regarding the management and evacuation of surgical smoke generated during surgical procedures. During HIPEC/peritonectomy procedures, more extensive peritoneal excision and increased intraoperative bleeding compared with standard colorectal surgery necessitate the use of high-energy electrocautery (200–300 W) to achieve adequate hemostasis during peritonectomy. In addition, these procedures are considerably longer than standard colorectal operations. Consequently, operating room personnel are exposed to larger quantities of surgical smoke for prolonged periods.

In earlier studies, it has been demonstrated that when electrocautery is used, higher amounts of certain polycyclic aromatic hydrocarbons and ultrafine particles with known carcinogenic properties are generated. This is primarily due to the high tissue temperatures achieved during peritonectomy (600–700 °C), whereas significantly lower temperatures are typically observed in standard surgical procedures (150–400 °C) [9,10,11]. The mutagenic effect produced by the thermal destruction of 1 g of tissue has been reported to be equivalent to that of 3–6 cigarettes [11]. It is known that ultrafine particles present in surgical smoke and aerosol can reach the alveoli of the lungs and cause pulmonary damage [12]. Research by Andreasson et al. [13] demonstrated that concentrations of ultrafine particles measured 3 cm from the surgeon’s mouth are significantly higher during peritonectomy compared with standard colorectal resections, but in this study the blood CO/MetHb levels were not measured.

Experimental exposure of laboratory animals to high concentrations of ultrafine particles has been associated with inflammatory lung injury, including interstitial pneumonia and extensive emphysema [14,15,16,17,18]. However, the exposure levels used in these studies were considerably higher than those encountered in clinical practice or in our study.

We observed statistically significant increases in COHb during HIPEC/peritonectomy with a median increase from 0.7% at baseline to 1.1% at the end of the procedure. Statistical significance was confirmed with a mixed-effects approach. This suggests that surgical smoke, even when smoke evacuation systems are used, may still be inhaled to some extent, resulting in an elevation of COHb levels, particularly during longer procedures. However, the observed values were not indicative of clinically significant CO toxicity, and none of the participants developed clinical symptoms attributable to CO exposure. The symptoms associated with such exposure are diverse and may include dizziness, paresthesia, chest pain, palpitations, abdominal pain, diarrhea, visual disturbances, headaches, flu-like symptoms, and chronic fatigue [19,20]. It is important to note that normal COHb levels do not exclude the diagnosis of chronic carbon monoxide exposure [21]. At the end, our findings should not be interpreted as evidence of clinically significant acute carbon monoxide poisoning or as demonstrating an immediate occupational health hazard. On the other hand, the long-term effect of exposure to surgical smoke has yet to be investigated. The main issue here is that reliable biochemical markers for chronic carbon monoxide exposure have not yet been established [22]. Our results can be explained by the fact that peritonectomy is a long surgical procedure (mean duration 10–12 h) during which high tissue temperatures are achieved, and the duration of electrocautery use is considerably extended.

Unlike COHb, the analysis of MetHb levels is somewhat difficult to interpret. Overall, these findings indicate that the between-group difference in MetHb was already present at baseline and persisted throughout the surgical procedure, while the magnitude of the difference gradually decreased over time. COHb is formed through competitive binding of carbon monoxide to hemoglobin, but MetHb is formed through the chemical oxidation of hemoglobin. Considering this mechanism, the risk of methemoglobinemia resulting from exposure to surgical smoke is generally considered negligible for operating room staff when all recommended technical and safety measures are appropriately implemented.

We could not identify the factors responsible for the higher baseline MetHb levels observed in some of our surgeons, as this was not an objective of the study and no specific tests were performed to investigate this phenomenon, which was only recognized during the final analysis. As reported, none of the participants were smokers, and no other occupational or personal-life exposures known to facilitate CO exposure were identified. The literature suggests that normal baseline MetHb levels are generally around 2%, although they may vary between individuals and can be chronically elevated due to genetic factors, lifestyle-related exposures, demographic characteristics, or underlying health conditions. Finally, we cannot conclude that exposure to surgical smoke during the studied procedures was associated with significant changes in MetHb levels, and no reliable clinical conclusions can be drawn from this part of the analysis.

Smoke evacuation systems positioned close to the source (2–5 cm) significantly enhance occupational safety; however, when placed further away, only approximately 50% of smoke is effectively evacuated [23,24,25]. A statistically significant increase in COHb levels in peripheral blood was observed in 25 patients who underwent endoscopic laser surgery in a study conducted by Ott et al. [26]. All patients had normal COHb levels before induction of anesthesia. The same study reported elevated MetHb levels in these patients, which ranged from baseline 1% to 2–3%. Wu et al. provided similar results in an animal model [27].

The production of surgical smoke elements depends on tissue type, patient demographic characteristics, duration of the procedure, and duration of electrocautery use. High-quality filtration masks or double masking may improve protection, but still do not provide adequate safety [28,29,30]. While they act as a barrier for larger particles (typically ≥5 µm), they are insufficient for smaller particulate matter [31,32].

Healthcare workers in operating theatres often do not adhere to established occupational health and safety protocols and frequently fail to use the recommended personal protective equipment [33]. This is largely attributable to a lack of interest among surgeons, insufficient support from employers, and the fact that smoke evacuation systems are not available. Employers’ commitment to workplace safety is crucial for operating room personnel, as it encourages greater awareness of occupational hazards and promotes safer working conditions [34,35,36].

We need to highlight that the available evidence regarding clinically significant systemic CO exposure in human surgeons remains limited and somewhat inconsistent. De Nezhat et al. prospectively studied 27 patients undergoing prolonged laparoscopic procedures with laser/electrosurgery and aggressive smoke evacuation. COHb did not increase meaningfully; only one patient had a postoperative COHb above 1%, and the authors concluded that CO poisoning was not associated with prolonged laparoscopic surgery under those conditions [37]. In addition, Hurst et al. published a review that demonstrated that the risks related to the surgical smoke have been overstated [38]. Hui et al. measured CO in the operating room during electrosurgery and found a high-smoke zone containing approximately 0.015% CO. In their experimental model, exposure to surgical smoke produced significantly higher COHb levels. They also evaluated a smoke evacuator, finding that evacuation reduced, but did not completely eliminate, the exposure [39]. During the 1990s, several studies investigated the effects of surgical smoke on COHb levels during laparoscopic procedures, demonstrating the potential of electrocautery to generate hazardous levels of CO, although not necessarily resulting in clinically significant systemic CO exposure or poisoning [40,41,42].

Kawaguchi et al. [43] conducted a randomized clinical trial involving 42 patients undergoing laparotomy. Smoke evacuation reduced acetaldehyde and formaldehyde by around 70%, and reduced particle counts by 80–95%, depending on particle size. Tyle et al. [44] also recommend safety measures, including smoke evacuators, because electrocautery smoke contains ultrafine particles and other potentially hazardous compounds, although they emphasize that evidence for actual human health effects remains incomplete.

During HIPEC/cytoreductive surgery/peritonectomy, extensive electrocautery and electro-evaporation of peritoneal tumor deposits may generate substantial amounts of surgical smoke over prolonged periods. This procedure has two different occupational hazards: the chemotherapy phase, which leads to cytotoxic drug exposure through skin contact, contaminated surfaces, aerosols/fumes, and potentially systemic absorption; and the cytoreductive/peritonectomy phase, which results in large amounts of surgical smoke. The recent HIPEC occupational safety literature focuses much more heavily on cytotoxic drug exposure than on CO from surgical smoke.

The newest systematic review from Fang et al. [45] suggests that measured occupational exposure to chemotherapy drugs during HIPEC is generally low when appropriate safety measures are used. However, surface contamination such as gloves, floors, and equipment remains an important exposure route. Double/triple gloves and fume-extraction systems were identified as effective preventive measures.

Sánchez-Carrillo et al. [10] specifically identified peritonectomy/electro-evaporation during HIPEC as producing large amounts of surgical smoke for prolonged periods. Their study suggests that the potential risks associated with surgical smoke, including CO exposure, should also be considered when establishing comprehensive occupational safety protocols. Ndaw et al. found substantial contamination of floors, gloves, shoes and devices, although urinary platinum was generally below quantification limits. The study suggests that dermal/contact exposure may be more important than inhalation exposure [46].

Despite ongoing technological advances and the development of new surgical devices, electrocautery remains the most widely used instrument for tissue cutting and coagulation. Newer technologies, such as ultrasonic scalpels and lasers, also generate surgical smoke; consequently, operating room personnel will continue to be exposed to the harmful effects of smoke inhalation in the future. This underscores the need for further research into the impact of surgical smoke exposure on the development of acute and chronic diseases.

### Limitations

In this study, direct environmental measurements of CO concentrations in the operating room were not performed. The primary aim of the study was to evaluate biological markers of exposure rather than to characterize the operating room environment itself or compare this to blood results. Also, adequate equipment for continuous environmental CO monitoring was not available. Therefore, although the observed changes in COHb and MetHb may support an association between the surgical procedure and exposure to smoke, the present study cannot directly quantify the corresponding environmental CO burden or establish a direct relationship between ambient CO concentrations and biological exposure. Second, there is a comparability issue, as the procedures studied differ in terms of their technical characteristics, complexity, and indications. Understandably, patients were not randomized, as the choice of procedure was based on established clinical guidelines and the individual eligibility criteria for each surgical approach. Thus, it is clear that HIPEC is employed in cases with a higher tumor stage and greater tumor burden, which inevitably results in longer operative times and more prolonged use of electrocautery. In this study, our aim was not to identify the specific factors contributing to changes in Co/MethHb levels. Rather, we sought to characterize the overall changes in Co/MethHb levels associated with each procedure and in relation to operative duration for each member of the surgical staff. The comparative part of the study, with regard to the type and duration of the procedure, was therefore a secondary analysis performed simply to demonstrate potential differences in Co/MethHb levels. We did not further investigate the specific factors that might account for these differences, such as the greater use of energy devices, local ventilation characteristics, increased surgical complexity, or blood transfusions. Such factors were not included as a predefined procedure-related covariate, so these were not systematically collected, but future studies should further investigate these factors, preferably using multivariable logistic regression to account for potential confounding variables. Third, we did not directly measure environmental CO concentrations, as our operating rooms were not equipped for such measurements. However, the operating rooms have all the necessary licenses and meet the required safety standards and prerequisites to prevent harmful CO accumulation. Therefore, we considered it unlikely that environmental CO exposure significantly affected the observed results.

## 5. Conclusions

The aim of this study was to determine the effects of exposure of operating room staff to surgical smoke, primarily carbon monoxide. Measured carboxyhemoglobin levels in the blood of all surgeons and assistants were higher during peritonectomy procedures compared to standard colorectal resections, which corresponds with the greater production of particles and smoke. Although our study showed a statistically significant difference in the percentage of carboxyhemoglobin in the blood of all operators during peritonectomy compared to the control group, values never reached levels which are generally associated with symptoms of acute carbon monoxide poisoning. However, the possibility of chronic CO poisoning should be considered. Given the currently limited evidence, further research is needed to better understand the harmful effects of surgical smoke and to evaluate preventive measures, including the effectiveness of different types of surgical masks.

## Figures and Tables

**Table 1:** Patient demographics.

Variable	Standard Resection		Peritonectomy		*p*-Value
Age, median (min–max)	64 (44–83)		56.0 (40–73)		0.020
Sex (Male/Female)	9/6		3/12		0.06
BMI (kg/m^2^) mean ± SD	24.7 ± 3.7		25.1 ± 4.2		0.47
Surgery type/indication	Anterior rectal resection	n = 5	Ovarian carcinoma	n = 7	
	Right hemicolectomy	n = 4	Colorectal carcinoma	n = 2	
	Left hemiolectomy	n = 3	Pseudomyxoma peritonei	n = 5	
	Sigmoidectomy	n = 3	Gastric adenocarcinoma	n = 1	

**Table 2:** COHb levels according to surgical procedure type.

	Surgery Type	C	25. P.	75. P.	Z	P	Effect Size r
COHb-0. min (%)	Standard resection	0.60	0.50	1.00	−1.217	0.224	0.222
	Peritonectomy	0.70	0.60	0.90			
COHb-20. min (%)	Standard resection	0.70	0.60	0.90	−6.057	<0.001	1.106
	Peritonectomy	1.00	1.00	1.20			
COHb-40. min (%)	Standard resection	0.70	0.40	0.80	−6.649	<0.001	1.214
	Peritonectomy	1.20	1.00	1.30			
COHb-end of procedure (%)	Standard resection	0.60	0.40	0.80	−6.559	<0.001	1.198
	Peritonectomy	1.10	1.00	1.20			

C—median, 25. P—25. percentile, 75. P.—75. percentile, Z—Mann–Whitney test value, P—statistical probability.

**Table 3:** MetHb levels according to surgical procedure type.

	Surgery Type	C	25. P.	75. P.	Z	P	Effect Size r
MetHb-0. min (%)	Standard resection	0.50	0.30	0.70	−4.464	<0.001	0.815
	Peritonectomy	0.20	0.10	0.30			
MetHb-20. min (%)	Standard resection	0.50	0.20	0.60	−3.077	0.002	0.562
	Peritonectomy	0.20	0.20	0.40			
MetHb-40. min (%)	Standard resection	0.40	0.20	0.50	−2.461	0.014	0.449
	Peritonectomy	0.30	0.20	0.30			
MetHb-end of procedure (%)	Standard resection	0.40	0.20	0.50	−2.265	0.024	0.414
	Peritonectomy	0.30	0.20	0.40			

C—median, 25. P—25. percentile, 75. P.—75. percentile, Z—Mann–Whitney test value, P—statistical probability.

**Table 4:** COHb levels in primary surgeon according to procedure type.

	Surgery Type	C	25. P.	75. P.	Z	P	Effect Size (r)
COHb-0. min (%)	Standard resection	0.60	0.50	1.10	−0.487	0.653	0.089
	Peritonectomy	0.70	0.60	0.80			
COHb-20. min (%)	Standard resection	0.70	0.40	1.00	−2.853	0.004	0.521
	Peritonectomy	1.00	1.00	1.20			
COHb-40. min (%)	Standard resection	0.70	0.40	0.90	−3.228	0.001	0.589
	Peritonectomy	1.10	1.00	1.30			
COHb-end of procedure (%)	Standard resection	0.70	0.40	0.80	−3.321	0.001	0.606
	Peritonectomy	1.00	1.00	1.10			

C—median, 25. P—25. percentile, 75. P.—75. percentile, Z—Mann–Whitney test value, P—statistical probability.

**Table 5:** MetHb levels in primary surgeon according to procedure type.

	Surgery Type	C	25. P.	75. P.	Z	P
MetHb-0. min (%)	Standard resection	0.40	0.10	0.50	−2.226	0.026
	Peritonectomy	0.20	0.10	0.20		
MetHb-20. min (%)	Standard resection	0.40	0.20	0.50	−1.411	0.158
	Peritonectomy	0.20	0.20	0.30		
MetHb-40. min (%)	Standard resection	0.30	0.20	0.50	−0.835	0.404
	Peritonectomy	0.30	0.20	0.30		
MetHb-end of procedure (%)	Standard resection	0.30	0.10	0.50	−0.663	0.507
	Peritonectomy	0.30	0.20	0.30		

C—median, 25. P—25. percentile, 75. P.—75. percentile, Z—Mann–Whitney test value, P—statistical probability.

**Table 6:** COHb levels in first assistant according to procedure type levels in first assistant.

	Surgery Type	C	25. P.	75. P.	Z	P
COHb-0. min (%)	Standard resection	0.80	0.50	1.10	−0.672	0.512
	Peritonectomy	0.80	0.70	1.00		
COHb-20. min (%)	Standard resection	0.70	0.50	0.90	−3.688	<0.001
	Peritonectomy	1.20	0.90	1.30		
COHb-40. min (%)	Standard resection	0.60	0.40	0.80	−4.165	<0.001
	Peritonectomy	1.20	1.10	1.40		
COHb-end of procedure (%)	Standard resection	0.60	0.30	0.70	−3.994	<0.001
	Peritonectomy	1.10	1.00	1.20		

C—median, 25. P—25. percentile, 75. P.—75. percentile, Z—Mann–Whitney test value, P—statistical probability.

**Table 7:** MetHb levels in first assistant according to procedure type.

	Surgery Type	C	25. P.	75. P.	Z	P
MetHb-0. min (%)	Standard resection	0.60	0.30	0.70	−1.896	0.058
	Peritonectomy	0.30	0.20	0.50		
MetHb-20. min (%)	Standard resection	0.50	0.20	0.60	−1.069	0.285
	Peritonectomy	0.30	0.20	0.50		
MetHb-40. min (%)	Standard resection	0.50	0.20	0.60	−0.987	0.323
	Peritonectomy	0.30	0.20	0.50		
MetHb-end of procedure (%)	Standard resection	0.50	0.20	0.50	−0.976	0.329
	Peritonectomy	0.30	0.20	0.50		

C—median, 25. P—25. percentile, 75. P.—75. percentile, Z—Mann–Whitney test value, P—statistical probability.

**Table 8:** COHb levels in second assistant according to procedure type.

	Surgery Type	C	25. P.	75. P.	Z	P
COHb-0. min (%)	Standard resection	0.60	0.50	0.90	−0.955	0.367
	Peritonectomy	0.70	0.60	0.90		
COHb-20. min (%)	Standard resection	0.70	0.60	0.80	−4.108	<0.001
	Peritonectomy	1.00	1.00	1.20		
COHb-40. min (%)	Standard resection	0.70	0.60	0.80	−4.251	<0.001
	Peritonectomy	1.20	1.00	1.30		
COHb-end of procedure (%)	Standard resection	0.70	0.50	0.80	−4.185	<0.001
	Peritonectomy	1.00	1.00	1.20		

C—median, 25. P—25. percentile, 75. P.—75. percentile, Z—Mann–Whitney test value, P—statistical probability.

**Table 9:** MetHb levels in second assistant according to procedure type.

	Surgery Type	C	25. P.	75. P.	Z	P
MetHb-0. min (%)	Standard resection	0.50	0.40	0.70	−3.661	<0.001
	Peritonectomy	0.20	0.10	0.40		
MetHb-20. min (%)	Standard resection	0.50	0.40	0.70	−2.888	0.004
	Peritonectomy	0.30	0.20	0.40		
MetHb-40. min (%)	Standard resection	0.50	0.30	0.60	−2.629	0.009
	Peritonectomy	0.30	0.20	0.40		
MetHb-end of procedure (%)	Standard resection	0.40	0.30	0.60	−2.192	0.028
	Peritonectomy	0.30	0.20	0.40		

C—median, 25. P—25. percentile, 75. P.—75. percentile, Z—Mann–Whitney test value, P—statistical probability.

**Table 10:** COHb levels in surgeons according ot duration of standard colorectal resections.

Surgery Type = Standard Colorectal Resection	C	25. P.	75. P.	χ^2^	P
COHb-0. min (%) (A)	0.60	0.50	0.90	23.855	<0.001
COHb-20. min (%) (B)	0.70	0.60	0.80		
COHb-40. min (%) (C)	0.70	0.40	0.70		
COHb-end of procedure (%) (D)	0.60	0.30	0.70		
Post hoc, Wilcoxon test	Z	P			
A:B	−1.910	0.056			
A:C	−2.344	0.019			
A:D	−3.081	0.002			
B:C	−2.169	0.030			
B:D	−3.958	<0.001			
C:D	−3.530	<0.001			

C—median, 25. P—25. percentile, 75. P.—75. percentile, Z—Mann–Whitney test value, P—statistical probability.

**Table 11:** MetHb in surgeons according to duration of standard colorectal resection.

Surgery Type = Standard Colorectal Resection	C	25. P.	75. P.	χ^2^	P
MetHb-0. min (%) (A)	0.50	0.20	0.70	37.655	<0.001
MetHb-20. min (%) (B)	0.50	0.20	0.60		
MetHb-40. min (%) (C)	0.40	0.20	0.50		
MetHb-end of procedure (%) (D)	0.40	0.20	0.50		
Post hoc, Wilcoxon test	Z	P			
A:B	−1.793	0.073			
A:C	−3.119	0.002			
A:D	−3.838	<0.001			
B:C	−2.824	0.005			
B:D	−3.579	<0.001			
C:D	−2.449	0.014			

C—median, 25. P—25. percentile, 75. P.—75. percentile, Z—Mann–Whitney test value, P—statistical probability.

**Table 12:** COHb in surgeons according to procedure duration.

HIPEC/Peritonectomy	C	25. P.	75. P.	χ^2^	P
COHb-0. min (%) (A)	0.70	0.60	0.80	84.504	<0.001
COHb-20. min (%) (B)	1.00	1.00	1.20		
COHb-40. min (%) (C)	1.20	1.00	1.20		
COHb-end of procedure (%) (D)	1.10	1.00	1.20		
Post hoc, Wilcoxon test	Z	P			
A:B	5.529	<0.001			
A:C	−5.857	<0.001			
A:D	−5.231	<0.001			
B:C	−3.263	<0.001			
B:D	−0.153	0.878			
C:D	−4.078	<0.001			

C—median, 25. P—25. percentile, 75. P.—75. percentile, Z—Mann–Whitney test value, P—statistical probability.

**Table 13:** MetHb in primary surgeon according to procedure duration.

HIPEC/Peritonectomy	C	25. P.	75. P.	χ^2^	P
MetHb-0. min (%) (A)	0.20	0.10	0.30	24.699	<0.001
MetHb-20. min (%) (B)	0.20	0.20	0.40		
MetHb-40. min (%) (C)	0.30	0.20	0.30		
MetHb-end of procedure (%) (D)	0.30	0.20	0.30		
Post hoc, Wilcoxon test	Z	P			
A:B	−2.908	0.004			
A:C	−3.448	<0.001			
A:D	−2.710	0.007			
B:C	−0.501	0.616			
B:D	−0.149	0.881			
C:D	−0.279	0.780			

**Table 14:** Comparison of the increase in COHb levels between studied groups.

Type of Surgery		C	25. P.	75. P.	Z	P
COHb levels increase difference	Standard resection	−0.20	−0.30	0	−6.096	<0.001
	HIPEC/Peritonectomy	0.30	0.10	0.50		

C—median, 25. P—25. percentile, 75. P.—75. percentile, Z—Mann–Whitney test value, P—statistical probability.

**Table 15:** Comparison of the increase in MetHb levels between surgical procedure types.

Type of Surgery		C	25. P.	75. P.	Z	P
MetHb levels increase difference	Standard resection	−0.10	−0.10	0	−4.845	<0.001
	HIPEC/Peritonectomy	0.10	0	0.10		

C—median, 25. P—25. percentile, 75. P.—75. percentile, Z—Mann–Whitney test value, P—statistical probability.

**Table 16:** Linear mixed-effects analysis of COHb.

**Time Point**	**SCRG, Mean ± SD (%)**	**HPG, Mean ± SD (%)**	**Between-Group Difference, β (95% CI)**	***p*-Value**	
0 min	0.76 ± 0.30	0.77 ± 0.14	−0.007 (−0.150 to 0.137)	0.928	
20 min	0.77 ± 0.18	1.10 ± 0.12	−0.333 (−0.477 to −0.190)	<0.001	
40 min	0.69 ± 0.25	1.25 ± 0.16	−0.560 (−0.704 to −0.416)	<0.001	
End of observation	0.66 ± 0.25	1.15 ± 0.12	−0.487 (−0.630 to −0.343)	<0.001	
**Fixed Effect**	**β Coefficient**	**SE**	**95% CI**	**z**	***p *** **Value**
Intercept (Standard, 0 min)	0.760	0.052	0.658–0.862	14.67	<0.001
Peritonectomy vs. Standard	0.007	0.073	−0.137–0.150	0.09	0.928
20 vs. 0 min	0.007	0.022	−0.036–0.049	0.31	0.758
40 vs. 0 min	−0.073	0.022	−0.116–−0.031	−3.39	0.001
End vs. 0 min	−0.100	0.022	−0.142–−0.058	−4.63	<0.001
Peritonectomy × 20 min	0.327	0.031	0.267–0.387	10.69	<0.001
Peritonectomy × 40 min	0.553	0.031	0.493–0.613	18.10	<0.001
Peritonectomy × End	0.480	0.031	0.420–0.540	15.70	<0.001

**Table 17:** Linear mixed-effects analysis of MetHb.

**Time Point**	**SCRG, Mean ± SD (%)**	**HPG, Mean ± SD (%)**		**Between-Group Difference, β (95% CI)**		***p*-Value**
0 min	0.21 ± 0.10	0.51 ± 0.21		0.305 (0.192–0.418)		<0.001
20 min	0.29 ± 0.14	0.45 ± 0.22		0.157 (0.041–0.273)		0.008
40 min	0.28 ± 0.09	0.38 ± 0.17		0.104 (−0.012–0.220)		0.078
End of observation	0.27 ± 0.10	0.38 ± 0.17		0.114 (−0.002–0.230)		0.054
**Fixed Effect**	**β**	**SE**	**95% CI**	**z**	***p*** **-Value**	
Intercept (Peritonectomy, 0 min)	0.205	0.041	0.125–0.285	5.035	<0.001	
Standard group	0.305	0.058	0.192–0.418	5.283	<0.001	
Time: 20 min	0.088	0.011	0.066–0.110	7.844	<0.001	
Time: 40 min	0.075	0.011	0.053–0.097	6.655	<0.001	
Time: End	0.065	0.011	0.043–0.087	5.764	<0.001	
Standard × 20 min	−0.148	0.016	−0.179 to −0.117	−9.328	<0.001	
Standard × 40 min	−0.201	0.016	−0.232 to −0.170	−12.689	<0.001	
Standard × End	−0.191	0.016	−0.222 to −0.160	−12.059	<0.001	

## Data Availability

The datasets presented in this article are not readily available due to technical/time limitations. Requests to access the datasets should be directed to first author.

## Abbreviations

The following abbreviations are used in this manuscript:

BMI	Body mass index
CO	Carbon monoxide
COHb	Carboxyhemoglobin
COVID-19	Coronavirus Disease of 2019
HPG	HIPEC-peritonectomy group
MetHb	Methemoglobin
ORP	Operating room personnel
SCRG	Standard colorectal resection group
SD	Standard deviation
